# Depressive symptoms, education, gender and history of migration - an intersectional analysis using data from the German National Cohort (NAKO)

**DOI:** 10.1186/s12939-025-02479-2

**Published:** 2025-04-21

**Authors:** Nico Vonneilich, Heiko Becher, Klaus Berger, Patricia Bohmann, Hermann Brenner, Stefanie Castell, Nico Dragano, Volker Harth, Stefanie Jaskulski, André Karch, Thomas Keil, Lilian Krist, Berit Lange, Michael Leitzmann, Janka Massag, Claudia Meinke-Franze, Rafael Mikolajczyk, Nadia Obi, Tobias Pischon, Marvin Reuter, Börge Schmidt, Ilais Moreno Velásquez, Henry Völzke, Christian Wiessner, Olaf von dem Knesebeck, Daniel Lüdecke

**Affiliations:** 1 Institute of Medical Sociology, University Medical Center Hamburg-Eppendorf, Hamburg, Germany; 2 Institute of Global Health, University Hospital Heidelberg, Heidelberg, Germany; 3 Institute of Epidemiology and Social Medicine, University of Münster, Münster, Germany; 4 Department of Epidemiology and Preventive Medicine, University of Regensburg, Regensburg, Germany; 5 Division of Clinical Epidemiology and Ageing Research, German Cancer Research Center (DKFZ), Heidelberg, Germany; 6 Department for Epidemiology, Helmholtz Centre for Infection Research, Brunswick, Germany; 7 Institute of Medical Sociology, Centre for Health and Society, University Hospital and Medical Faculty, Heinrich Heine University Düsseldorf, Düsseldorf, Germany; 8 Institute for Occupational and Maritime Medicine (ZfAM), University Medical Center Hamburg-Eppendorf, Hamburg, Germany; 9 Institute for Prevention and Cancer Epidemiology, Faculty of Medicine and Medical Center, University of Freiburg, Freiburg, Germany; 10 Institute of Social Medicine, Epidemiology and Health Economics, Charité-Universitätsmedizin Berlin, Berlin, Germany; 11 Institute of Clinical Epidemiology and Biometry, University of Würzburg, Würzburg, Germany; 12 State Institute of Health I, Bavarian Health and Food Safety Authority, Erlangen, Germany; 13 Institute for Medical Epidemiology, Biometrics, and Informatics, Interdisciplinary Center for Health Sciences, Martin Luther University Halle-Wittenberg, Halle, Germany; 14 Institute for Community Medicine, University Medicine Greifswald, Greifswald, Germany; 15 German Center for Mental Health (DZPG), Halle, Germany; 16 Center for Intervention and Research on adaptive and maladaptive brain Circuits underlying mental health (C-I-R-C), Magdeburg, Germany; 17 Institute of Medical Biometry and Epidemiology, University Medical Center Hamburg-Eppendorf, Hamburg, Germany; 18 Molecular Epidemiology Research Group, Max-Delbrueck-Center for Molecular Medicine in the Helmholtz Association (MDC), Berlin, Germany; 19 Max-Delbrueck-Center for Molecular Medicine in the Helmholtz Association (MDC), Biobank Technology Platform, Berlin, Germany; 20 Charité - Universitätsmedizin Berlin, corporate member of Freie Universität Berlin and Humboldt- Universität zu Berlin, Berlin, Germany; 21 Junior Professorship for Sociology, esp. Work and Health, Department of Sociology, University of Bamberg, Bamberg, Germany; 22 Institute for Medical Informatics, Biometry and Epidemiology, University Hospital of Essen, Essen, Germany

**Keywords:** German national cohort, NAKO, Intersectional analysis, Educational inequalities, Depression, History of migration, Gender, MAIHDA

## Abstract

**Background:**

The educational gradient in depressive symptoms is well documented. Gender and history of migration have also been found to be associated with depressive symptoms. Intersectional approaches enable the analysis of the interplay of different social factors at a time to gain a deeper understanding of inequalities in depressive symptoms. In this study, intersectional inequalities in depressive symptoms according to education, gender and history of migration are analysed.

**Methods:**

The German National Cohort (NAKO, *N* = 204,783) collected information on depressive symptoms (PHQ-9), which was used as an outcome variable. Educational attainment (ISCED-97), gender, and history of migration constituted the different social strata in the analyses. The predicted probabilities of depressive symptoms for 30 social strata were calculated. Multilevel analysis of individual heterogeneity and discriminatory accuracy (MAIHDA) was applied, using logistic regression and social strata were introduced as higher-level unit interaction terms.

**Results:**

The analyses revealed an educational gradient in depressive symptoms, with differences within each educational group when gender and history of migration were introduced to the models. The predicted probabilities of depressive symptoms varied between the most advantaged and the most disadvantaged social strata by more than 20% points. Among the three studied variables, education contributed the most to the variance explained by the MAIHDA models. The between-strata differences were largely explained by additive effects.

**Conclusions:**

We observed a robust educational gradient in depressive symptoms, but gender and history of migration had substantial contribution on the magnitude of educational inequalities. An intersectional perspective on inequalities in depressive symptoms enhances current knowledge by showing that different social dimensions may intersect and contribute to inequalities in depressive symptoms. Future studies on inequalities in depression may greatly benefit from an intersectional approach, as it reflects lived inequalities in their diversity.

**Supplementary Information:**

The online version contains supplementary material available at 10.1186/s12939-025-02479-2.

## Background

The educational gradient of depressive symptoms is well documented by extensive research: the lower the educational attainment is, the greater the risk of experiencing depressive symptoms [1–3]. As such, education can be understood as a fundamental cause of mental health outcomes, including depressive symptoms [4]. Among the components explaining the educational gradient are poorer coping mechanisms, behaviour-related risk factors, stress exposure, critical life events, a lower sense of control, fewer material resources, and lower access to health care for people with lower educational attainment [2, 5]. However, studies on educational inequalities in depressive symptoms have been criticized for neglecting within-group heterogeneity and focusing mostly on single-axis inequalities [6, 7]. These methods and analyses might not reveal the interplay of different social categories, as there are several other inequalities in depressive symptoms at the population level, such as inequalities by age, gender, history of migration, or ethnicity [8–10]. The interaction of these social categories may affect the educational gradient of depressive symptoms.

In earlier research, gender and a history of international migration were repeatedly shown to be associated with depressive symptoms. With respect to gender and depressive symptoms, higher risks have consistently been detected in women than in men [4, 7–13]. On the basis of an international meta-analysis of data from more than 1.7 million women and men, Salk and colleagues reported significantly increased odds of depression in women compared with men [14]. These differences varied across the lifespan, with a peak in adolescence, but remained relatively stable across later adulthood. When examining associations of history of migration and depressive symptoms, international studies point towards higher rates in migrant populations. In their overarching review of reviews, Close et al. reported increased risks of poor mental health for first-generation migrants but reported wide variation in prevalence [15]. In terms of a potential healthy migrant effect in mental health, there may be a mental health advantage for some immigrants at time of arrival (younger, male), but most of the evidence shows a decline in mental health after immigration and higher mental health risks in migrant populations [16]. Few studies have investigated the associations between a history of migration and depressive symptoms in Germany [9, 17–19]. In a first publication based on data from the German National Cohort (NAKO), we identified greater depressive risks for migrant populations than for non-migrant populations in Germany [10]. Similar results were found in analyses based on a European sample [20].

The benefit of an intersectional approach is to analyse interactions at the intersections of privilege and disadvantage to gain a comprehensive understanding of inequalities in depressive symptoms. It goes back to black feminist movements in the U.S., specifically the Combahee River Collective and later Kimberlé Crenshaw [21, 22]. The experiences of black women being subjected to multiple forms of discrimination and inequality led to the formation of intersectionality to analyse the structures produced by interlocking systems of privilege and disadvantage (sexism, ageism or racism [23]). The intersectionality approach is increasingly applied, as it emphasizes multidimensional aspects of inequality and the potential multiplicative effects of different disadvantages [23].

In their review, Patil et al. reported that there are only a few studies on intersectional inequalities in depressive symptoms, and they have focused mainly on the U.S [6]. Evans and Erickson [7] applied a new approach in intersectionality research, i.e., the multilevel analysis of individual heterogeneity and discriminatory accuracy (MAIHDA). Their analysis revealed that different social strata, identified by gender, ethnicity, and income, differed substantially from each other in terms of depressive risks. Studies on intersectional inequalities in depressive symptoms in Germany are lacking. Wandschneider and colleagues analysed subjective mental health along the intersections of sex, gendered practices, and history of migration on the basis of German panel data but did not include indicators of socioeconomic status such as income or education [24]. In their review, Trygg and colleagues [25] identified only one European study that analysed health outcomes at the intersections of gender, household income, and country of birth on the basis of Swedish data [26]. As most of the evidence up to date stems from the US, the transferability and comparability of these results is difficult, especially because of differences in educational systems, social stratification, and differing categories concerning the history of migration in European countries compared with the US.

Based on data from the NAKO, the aim of the present paper is to analyse the variations within the educational gradient in depressive symptoms when gender and history of migration are simultaneously considered. The following research questions will be addressed via an intersectional approach: Are there variations within the educational gradient in depressive symptoms according to gender and history of migration in Germany? Do the intersectional strata significantly differ in terms of depressive symptoms? Do education, gender, and history of migration interact, indicating intersectional inequalities in depressive symptoms?

## Methods

### Data

The NAKO is a prospective multicentre cohort study in Germany [27]. The main goal of the NAKO is to investigate risk factors and causes of common chronic diseases. The study design was described elsewhere [27]. In brief, data collection and assessment are organized through 18 study centres, covering rural and urban areas [28–30]. The baseline assessment took place from March 2014 until September 2019 and included standardized face‒to‒face interviews, self-administered questionnaires, various physical examinations, and the collection of biospecimens. The NAKO included 204,783 participants aged 19–74 years at baseline, selected on the basis of random samples from the population registration authorities of the respective study locations, stratified by sex (1:1) and age (10.0% each 10-year group between 20 and 39 years and 26.7% in each 10-year group between 40 and 74 years). The overall response at baseline was 17%, but it varied between 9% and 32% across the study centers [31]. Further details on the sampling and data assessment can be found elsewhere [28, 29]. All participants provided written consent for study participation, and the study centers’ local ethical committees gave their approval. The complete study was carried out in accordance with the Declaration of Helsinki.

### Measures

#### Dependent variable

Depressive symptoms were assessed via the Patient Health Questionnaire (PHQ-9) [32], a well-established measure that has also been used in recent NAKO analyses [3, 10, 33]. The PHQ-9 consists of nine items asking about depressive symptoms in the past two weeks. Each item has a four-point scale ranging from 0 (“not at all”) to 3 (“almost every day”). A sum score ranging from 0 to 27 was calculated. The Cronbach’s alpha for the PHQ-9 scale was 0.84, indicating the high internal consistency of the scale. A validated cut-off score of 10 points or higher indicates depressive symptoms in terms of a moderate to severe depressive episode [34]. A binary variable derived from the PHQ-9 score (*≥* 10 vs. <10) was used in the present analyses for two reasons. First, the PHQ-9 score is heavily right skewed, which is why we cannot use a simpler, linear model. Second, we aimed to identify relevant depressive symptoms and therefore we did not use the mean value of the PHQ-9 score. The mean value is approximately 5, with a standard deviation of approximately 3.5, which means that people with moderate to severe depressive symptoms (PHQ-9 score *≥* 10) are lost in the overall sample.

#### Independent variables

Education was used as an indicator for the socioeconomic status and was assessed on the basis of the International Standard Classification of Education (ISCED-97), which is recoded into three levels (“low” = ISCED-level 1/2, “intermediate” = ISCED-level 3/4 and “high” = ISCED-level 5/6) [35].

History of migration was assessed according to a set of basic indicators for mapping migrant status in Germany [36], as proposed by Wiessner and colleagues for the NAKO data [30]. History of migration was recorded on the basis of the nationality and country of birth of both the study participants and their parents. This enabled a distinction of five subgroups: those without a history of migration, two subgroups of first-generation migrants with personal experience of migration (those with German citizenship (naturalized) and those without German citizenship), the group of German resettlers (a group of migrants from the former Soviet Union with German ancestors) and descendants of migrants (born in Germany having at least one parent not born in Germany) [30].

The gender of the respondents was recorded. On the basis of the numbers of subgroups of education (three), gender (two), and history of migration (five), 30 social strata were calculated. Age was introduced as a control variable (19–39 years, 40–59 years, 60 years and older).

#### Missing data

On average, approximately 16% of the individual items across all the variables were missing. The variables gender and age had no missing values, while the highest proportion of missing values was found for the variables education (9.1%) and PHQ-9 (7.5%). The missing data pattern was analysed, and missing data were imputed via multivariate imputation by chained equations method, generating five imputed datasets [37]. The outcome (PHQ-9) was included for imputation, as recommended by several authors [37–39]. Strata variables were included as regular terms, not their interaction. The method for imputing missing values depends on the variable’s nature. For continuous variables, predictive mean matching was applied, logistic regressions were used for binary variables, and polytomous logistic regression was used for categorical variables with more than two levels.

Comparisons of results based on the non-imputed data including missing values and the imputed data sets can be found in the supplementary material (see S1 and S2).

### Statistical analyses

In this study, we used the multilevel analysis of individual heterogeneity and discriminatory accuracy (MAIHDA), which is a statistical approach in social epidemiology that has increasingly gained attention in recent years. It is based on multilevel modelling where the different social strata are specified as higher-level units (“random effects”) [23]. This means that the estimated outcome in different social strata is “shrunken”, i.e., the (often too high) effects for smaller strata are pulled towards a global average. This is a desired property of multilevel models to protect against bias due to the ‘small N problem’ [40], which relates to single-level regression models, where predictors of social strata are entered as interaction terms. Larger numbers of strata can lead to strata that only have few observations, even in large datasets. These “outliers” result in biased estimates for strata on the basis of relatively few cases.

In our analysis, we used logistic regression multilevel models with “depressive symptoms” as the outcome. Social strata, defined by gender, education, and history of migration, were used as higher-level unit interaction terms (“random intercepts” on level 2) in all the models, whereas age was used as a control variable at the individual level (“fixed effect”).

The applied MAIHDA approach [23] comprises several steps. First, a “base model” with random effects only was fitted, with no further covariates. The goal was to summarize the overall inequality in the sample by calculating the intraclass correlation coefficient (ICC). The ICC ranges from zero to one and can be interpreted as the proportion of variance explained by the groups used as higher-level units [41]. The higher the ICC value is, the more the social strata contribute to the variance observed in the sample. An ICC close to zero would mean that belonging to a certain social stratum is not associated with the inequalities under study.

Next, additional multilevel models (“main effects models”) were fitted, one for each dimension (gender, education, and migration history) used to construct the social strata. The variables representing each dimension were separately added as predictors at the individual level, while the random effects remained unchanged. This step in the MAIHDA framework affects the resulting models’ ICC values and allows the calculation of the extent to which each dimension has an additive effect on inequalities, which is called the proportional change in variance (PCV, ranging from 0 to 1). The larger the PCV is, the greater the contribution of one dimension of the social strata to the overall inequalities (see also [42, 43].

In the final multilevel model (“full model”), all three dimensions were added as predictors on an individual level to assess whether there are simple additive effects regarding inequalities or whether there is an indication of interaction. The PCV will be nearly one when inequalities due to social strata are attributable to additive effects of gender, education, or migration history only. When the PCV is considerably lower than one, the remaining proportion of the total variance can be attributed to the interaction effects of the observed inequalities between strata.

To visualize the results and to determine how strongly each of the 30 different social strata (5 × 2 × 3) was associated with the outcome, ranked predicted probabilities were calculated, which illustrated the range, spread, and pattern of inequalities between strata. These estimate the adjusted probabilities of the “presence of depressive symptoms” for each stratum. As we focus on the potential of intersectional analysis for analysing the variability within educational inequalities in depressive symptoms, we visually emphasized the ranked predicted probabilities by education for each social stratum under study. Pairwise comparisons between the predicted probabilities were calculated within each level of education to test whether differences in inequalities between strata are statistically significant. Since all combinations of pairwise comparisons would result in very large tables, we focused on comparisons of strata against the group with the highest probability of depressive symptoms within each educational level.

All analyses were carried out via R statistical software and the packages glmmTMB, performance and ggeffects [44–46]. The related R code is deposited at osf.io (DOI: 10.17605/OSF.IO/C58GT).

## Results

**Table 1:** Depressive symptoms (PHQ-9), cut-off score (≥ 10), according to social dimensions and covariables; German National cohort (NAKO), *N* = 204,783

	% in the sample	% PHQ-9 > = 10 (95% CI)	*p* Value*	
**Total**		7.4 (7.3, 7.5)		
**Education**			< 0.001	
Low	2.7	13.9 (13.0, 14.9)		
Intermediate	42.2	9.0 (8.9, 9.2)		
High	55.1	5.8 (5.6, 5.9)		
**Gender**			< 0.001	
Men	49.5	5.9 (5.8, 6.1)		
Women	50.5	8.8 (8.6, 9.0)		
**History of migration**			< 0.001	
Non-migrants	83.1	6.9 (6.8, 7.1)		
1st generation migrants, without German citizenship	5.1	9.2 (8.6, 9.7)		
1st generation migrants, German citizenship	5.2	9.8 (9.2, 10.4)		
Descendants of migrants	5.0	9.7 (9.1, 10.3)		
Resettlers	1.7	9.0 (8.1, 10.0)		
**Age groups**			< 0.001	
< 40	20.3	8.8 (8.5, 9.1)		
40–59	52.7	8.1 (7.9, 8.3)		
≥ 60	27.0	4.9 (4.7, 5.1)		

*Chi-square test

In Table 1, an overview of all variables used in the present analyses is shown. A higher prevalence of depressive symptoms (PHQ-9 score of 10 or higher) was found in groups with low educational attainment (13.9%), in women (8.8%), and in groups with a history of migration. Depressive symptoms were more often reported by persons under the age of 40 years.

In Table 2, the fixed effects of the MAIHDA logistic regression models are reported. In Model 1, age was introduced as a covariate. The ICC for the basic model indicated that approximately 5% of the explained variance can be accounted for by the random effects of history of migration, gender, and education. The introduction of fixed effects by history of migration decreased the explained variance at level 2 (ICC 0.047), indicating a minimal effect of higher-level variation by history of migration (PCV = 0.094). The odds ratios for depressive symptoms were increased in 1st generation migrants with German citizenship and descendants of migrants. The introduction of gender as an additional fixed effect decreased the ICC by approximately 0.01, similarly indicating an effect of higher-level variation by gender (PCV = 0.185). In Model 4, the educational gradient in depressive symptoms became visible, i.e., lower educational attainment was associated with increased odds for depressive symptoms. The ICC was reduced by 0.02; likewise, a PCV of 0.615 indicated a high share of explained variance attributable to education. In Model 5 where all fixed effects were accounted for, the educational gradient in depressive symptoms remained significant and similar effects of gender and history of migration were detected as in Models 2 and 3. The ICC was low at 0.007 and the PCV of 0.874 indicated a high share of explained variance attributable to the fixed effects in the model. As indicated by the PCV of 0.87, there remains some evidence for interactive effects.

**Fig. 1:**
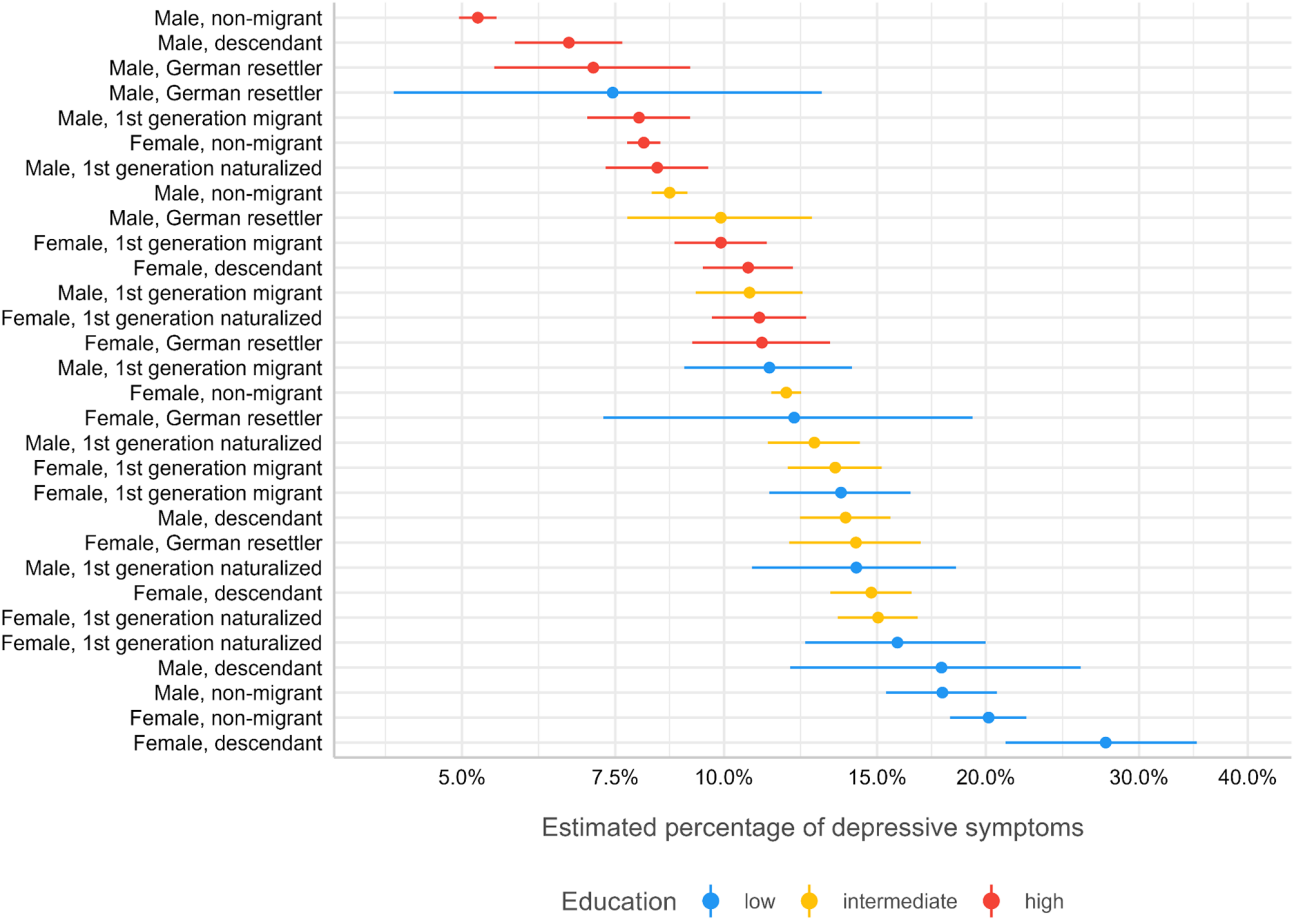
Ranked predicted probabilities (95% CI) of depressive symptoms (PHQ-9, cut-off score > 10) for 30 different social strata on the basis of education, gender, and history of migration; based on MAIHDA logistic regression Model 1; German National Cohort (NAKO), *N* = 204,783

To assess the magnitude of inequalities in depressive symptoms according to education, gender, and history of migration, the predicted probabilities of depressive symptoms for each of the 30 social strata were calculated based on Model 1. Figure 1 presents the distribution of probabilities of depressive symptoms, including 95% confidence intervals. The social stratum of highly educated and non-migrant males had the lowest predicted probability of depressive symptoms. The probabilities of most other social strata followed an educational gradient. There were few exceptions to this pattern, such as non-migrant males with intermediate educational levels or male first-generation migrants with low education levels. Within each educational stratum, considerable variance was observed. For example, in the low-educated strata (Fig. 1, blue), there was a difference of approximately 20% points, varying from 7% probability in low-educated male German resettlers to approximately 28% probability of depressive symptoms in low-educated female descendants. In general, women and those with a history of migration had higher probabilities of depressive symptoms than men and those without a history of migration within each educational stratum.

To detect the significance of these variations, as presented in Fig. 1, pairwise comparisons were performed, with the group with the highest probability of depressive symptoms within each educational stratum used as the reference group (see Table 3–4). In the high educational stratum, differences varied between 0 and 6% points and were therefore smaller than the differences observed in the lowest educational stratum (Table 3). Five of these differences in the high educational stratum were statistically significant. Within the intermediate educational stratum, the observed differences varied between 0 and 6% points, with largest difference for non-migrant males compared to naturalized female migrants (Table 5). Four out of nine observed differences reached statistical significance. Within the low educational stratum, differences varied between 7 and 20% points. Largest difference was observed for male German resettlers compared to female migrant descendants and one out of nine differences in the lowest educational stratum did not reach statistical significance (Table 4).

Results in the Tables and in Fig. 1 showed strongest associations between education and depressive symptoms. This is in line with the calculated proportional change in the between stratum variance in the MAIHDA logistic regression models (see Table 2). Education had the strongest impact on the explained variance in the model (PCV = 0.62), followed by gender (PCV = 0.19) and history of migration (PCV = 0.09).

**Table 2:** History of migration, gender, education, and depressive symptoms (PHQ-9, cut-off score > 10), MAIHDA logistic regression models, adjusted for age; German National cohort (NAKO), *N* = 204,783

Parameter	Model 1	Model 2	Model 3	Model 4	Model 5
	OR (95% CI)	OR (95% CI)	OR (95% CI)	OR (95% CI)	OR (95% CI)
**(Intercept)**	0.13 (0.11, 0.15)	0.12 (0.09, 0.17)	0.11 (0.09, 0.13)	0.09 (0.08, 0.11)	0.07 (0.06, 0.08)
**Age (ref. < 40 years)**	1.00	1.00	1.00	1.00	1.00
40–59 years	0.91 (0.87, 0.94)	0.91 (0.87, 0.94	0.91 (0.87, 0.94	0.90 (0.87, 0.94	0.90 (0.87, 0.94
60 + years	0.52 (0.49, 0.55)	0.52 (0.49, 0.55)	0.52 (0.49, 0.55)	0.52 (0.49, 0.55)	0.52 (0.49, 0.55)
**History of migration (ref. non-migrants)**		1.00			1.00
1st generation migrants		1.01 (0.63, 1.60)			1.04 (0.86, 1.26)
1st generation migrants, naturalized		1.18 (0.74, 1.88)			1.24 (1.02, 1.50)
Descendants of migrants		1.36 (0.85, 2.18)			1.35 (1.11, 1.64)
German resettler		0.86 (0.53, 1.41)			1.04 (0.83, 1.31)
**Gender (ref: men)**			1.00		1.00
Women			1.43 (1.07, 1.90)		1.39 (1.22, 1.58)
**Education (ref: high)**				1.00	1.00
Low education, ISCED-level 1 or 2				2.14 (1.65, 2.78)	2.23 (1.87, 2.66)
Intermediate education, ISCED-level 3 or 4				1.56 (1.23, 1.98)	1.57 (1.35, 1.81)
***ICC*** ^***1***^	0.051	0.047	0.042	0.020	0.007
***PCV*** ^***2***^ ***(Model 1 compared to all others)***	-	0.094	0.185	0.615	0.874

^1^ Intraclass Correlation Coefficient

^2^ Proportional Change in Variance

**Table 3:** Pairwise comparison within the highly educated social stratum on the basis of ranked predicted probabilities of depressive symptoms (PHQ-9, cut-off score > = 10) by gender and history of migration; German National cohort (NAKO), *n* = 112,755

High education	Contrast^1^, %-points	95% CI	*p*
*German resettler-female*	*Ref.*	*Ref.*	
Non-migrant-male	-0.06	-0.08, -0.04	< 0.001
Descendant-male	-0.04	-0.07, -0.02	< 0.001
German resettler-male	-0.04	-0.07, -0.01	0.004
1st generation migrant-male	-0.03	-0.05, -0.01	0.009
1st generation naturalized-male	-0.03	-0.05, 0.00	0.024
Non-migrant-female	-0.03	-0.05, -0.01	0.004
1st generation migrant-female	-0.01	-0.03, 0.01	0.343
Descendant-female	0.00	-0.03, 0.02	0.745
1st generation naturalized-female	0.00	-0.03, 0.02	0.952

^**1**^ Contrasts indicate differences in %-points in relation to the reference group

**Table 4:** Pairwise comparison within the low-educated social stratum on the basis of ranked predicted probabilities of depressive symptoms (PHQ-9, cut-off score > = 10) by gender and history of migration; German National cohort (NAKO), *n* = 5,540

Low education	Contrast^1^, %-points	95% CI	*p*
*Descendant-female*	*Ref.*	*Ref.*	
German resettler-male	-0.20	-0.28, -0.12	< 0.001
1st generation migrant-male	-0.16	-0.24, -0.09	< 0.001
German resettler-female	-0.15	0.06, 0.25	< 0.001
1st generation migrant-female	-0.14	-0.21, -0.06	< 0.001
1st generation naturalized-male	-0.13	-0.21, -0.05	0.001
1st generation naturalized-female	-0.12	-0.20, -0.04	0.004
Descendant-male	-0.10	-0.19, 0.00	0.052
Non-migrant-male	-0.10	-0.17, -0.02	0.011
Non-migrant-female	-0.07	-0.15, 0.00	0.048

^**1**^ Contrasts indicate differences in %-points in relation to the reference group

**Table 5:** Pairwise comparison within the intermediate-educated social stratum on the basis of the predicted probabilities of depressive symptoms (PHQ-9, cut-off score > = 10) according to gender and history of migration; German National health study (NAKO), *n* = 86,488

Intermediate education	Contrast^1^, %-points	95% CI	*p*
*1st generation naturalized-female*	*Ref.*	*Ref.*	
Non-migrant-male	-0.06	-0.08, -0.05	< 0.001
German resettler-male	-0.05	-0.08, -0.02	< 0.001
1st generation migrant-male	-0.04	-0.07, -0.02	< 0.001
Non-migrant-female	-0.03	-0.05, -0.02	< 0.001
1st generation migrant-female	-0.02	-0.04, 0.01	0.171
1st generation naturalized-male	-0.02	-0.05, 0.00	0.039
German resettler-female	-0.01	-0.02, 0.04	0.568
Descendant-male	-0.01	-0.04, 0.01	0.291
Descendant-female	-0.00	-0.02, 0.03	0.816

^**1**^ Contrasts indicate differences in %-points in relation to the reference group

## Discussion

On the basis of data from the NAKO and logistic MAIHDA models, estimates for depressive symptoms in 30 different social strata, which were defined by educational level, gender and history of migration, were calculated. Similar to previous findings in the field, the analyses revealed an educational gradient in depressive symptoms [1–4]. Adding new knowledge to the field, the identified educational gradient varied substantially by gender and history of migration. The MAIHDA approach revealed that within each educational stratum, differences in the probability of depressive symptoms existed. For example, women with a history of migration tended to show more depressive symptoms within each educational stratum. The social stratum with the lowest probability of depressive symptoms was highly educated non-migrant males. The probabilities for depressive symptoms varied between the least and the most disadvantaged social strata by more than 20%. The analyses also revealed that education contributed more to the variance explained by the MAIHDA models than did gender and history of migration. Additive effects largely explain the variance between the social strata, but there remains some evidence for interactive effects, meaning that the calculated odds in the full model exceed the expected values in some cases. However, evidence for interactive effects is rather weak, we did not identify a clear pattern of interaction and we cannot rule out potential effects of data imputation. In order to assess these effects, we added a comparison of the results based on imputed and non-imputed data in the supplementary material (see supplementary material S3-S5).

A critique of analytic approaches in risk-factor epidemiology is the focus on variability between different social categories while neglecting the variability within and potential overlap of these categories. Thereby these strategies potentially overlook the interdependence of social factors and social strata [47, 48]. As our analyses revealed, the characteristics of other social factors, such as gender or history of migration, can have a significant effect on how pronounced educational inequalities in depressive symptoms ultimately turn out to be, even within different educational groups. The combinations of social dimensions can be more relevant for the outcome than the social dimension itself; by combining two or more social dimensions, the probabilities of depressive symptoms can exceed the originally assumed risk of these social dimensions. According to our results, the interaction of education and gender seems to be especially relevant, as men can be found more often in the lower risk probabilities for depressive symptoms, as well as within lower educational backgrounds, than women.

When comparing the three social factors that were used to identify the different social strata, education had the strongest effect on depressive symptoms, followed by gender and history of migration. This is in line with previous findings showing the magnitude of socioeconomic inequalities in depressive symptoms and mental health [1, 7, 49–51]. A number of known risk factors for depressive symptoms, such as psychosocial stressors, behavioural risk factors, limited resources, and lower access to health care, are more likely to occur in groups with lower educational backgrounds and can help explain these findings [49, 52].

Similar to a work by Evans and colleagues on depression in adolescence and early adulthood [7], we found substantial differences between different social strata and their respective risks of depressive symptoms, and in some cases, these differences exceeded the expected additive effects. Evans and colleagues found interactive effects in their U.S. cohort study on depression in adolescents and young adults, especially for being female and Latin or Native American [7]. They focused on family income, gender, race/ethnicity and immigration history, while in our study education, gender and history of migration were used. The significant differences which we identify in our analyses within educational strata, especially within the lower educated, showed that the mental health consequences of being a woman with a history of migration and a low level of education were quite different from those experienced by a man with low levels of education and without a history of migration. The different social strata need to be understood as unique positions within society with unique experiences in everyday life. This important lesson can be learned from an intersectional perspective on educational inequalities in depressive symptoms [7].

Descendants of migrants are especially vulnerable to depressive symptoms within the lowest educational strata (see also 10). Compared to other groups with a history of migration, descendants are less likely to experience language barriers or barriers related to citizenship or residence status. It is more likely that a stronger desire for social acceptance and disappointed hopes for educational careers, especially in comparison to the previous generation of immigrants, translates into greater risks of depressive symptoms [53–56]. A longer duration of residence in Germany has been found to be associated with worse mental health outcomes [57]. Moreover, constant attribution as an immigrant and recurrent experiences of stigma and discrimination might play a role in explaining greater mental health risks [9].

### Strengths and limitations

We limited the analyses to three social dimensions and 30 social strata on the basis of previous knowledge of the social risk factors for depressive symptoms [10, 12, 58] to ensure that the analyses were comprehensible. Intersectional analyses are, to a certain degree, limited by data capacity; therefore, only a finite number of social strata can be applied.

Earlier studies have noted that variance in intersectional MAIHDA analysis is often low overall. The share of variance that is explained by between-strata differences does not exceed 5% in our analyses, which is comparable to the findings of similar previous studies [7]. Small ICC values in logistic regression models do not necessarily indicate negligible effects [59]. In order to investigate in how far the binary outcome in the logistic regression models resulted in small ICC values, we also conducted a linear mixed model analysis using a log-transformed outcome (see supplementary material S6-S8). The results corroborated the logistic regression model, indicating no significant underperformance.

The overall response in NAKO was approximately 17%, even though representative sampling schemes were applied (see also [30, 60]. Responses vary considerably across different NAKO study centres [29, 31]. The study population differs from the general population, as seen in the underrepresentation of low-educated groups, making up only approximately 2% of the total NAKO sample. The smaller sample size in low-educated groups needs to be taken into account when variations in the probability of depressive symptoms are interpreted. It is likely that this leads to an underestimation of depressive risks in low educated groups and that the calculated estimates are also less precise due to the lower number of cases.

A further selection effect cannot be ruled out when looking at history of migration. For those participants with limited knowledge of the German language, measures to increase participation, such as home visits in some study areas, have been taken [61]. But the study language of the NAKO is German, and other languages or translations are not provided, limiting its participation to those with sufficient German language skills [60]. These selection effects led to an underrepresentation of marginalized groups [19, 62], especially those lacking officially registered addresses or sufficient resources for participating in the NAKO study. This may in turn be associated with an underestimation of depressive risk as well as related inequalities.

A limitation concerns the assessment of gender in the NAKO. This approach is limited to biological sex, as diverse genders, self-assessed genders, gender practices or sexual orientations were not assessed in the study [63].

There is a chance of underestimation of depressive symptoms in men compared with women in the sample. The standard instruments used in the NAKO, such as the PHQ-9, might not adequately reflect masculine norms and their effects on the assessment of depression and depressive symptoms [64]. On the other hand, the PHQ-9 has been evaluated to assess depression similarly in men and women in the general population [65].

A strength of the MAIHDA approach presented here is the visualization of the findings. The visualizations of the different social strata and their relative risks of depressive symptoms (see Fig. 1) revealed the variability of risks within a single social stratum, such as low education. Considering this within-group variation and finding analytic ways to make these variations visible is a major benefit of the MAIHDA approach in analysing intersectional inequalities in depressive symptoms [23]. When probabilities of depressive symptoms are calculated on the basis of the MAIHDA approach, no reference category is needed, where either the most privileged or marginalized groups are used as a reference to which all others are compared. Here, we were able to assess the probabilities of depressive symptoms in different social strata without referring to a specific group. Moreover, in MAIHDA social strata are compared to the population grand mean (see 59).

## Conclusions

Intersectionality as a framework for analysing social inequalities in depressive symptoms can be valuable, as it helps in understanding the multiple forms of advantages and disadvantages when different social dimensions are considered simultaneously. Our results reveal diversity within the educational gradient in depressive symptoms when gender and history of migration are added as social dimensions, resulting in 30 different social strata. We also find evidence that the specific combinations of these social dimensions matter for the risk of depressive symptoms. For example, within the low educated strata, which generally show higher risks of depressive symptoms compared to those with intermediate or high education, it matters if one is a woman and a descendant of migrants or a non-migrant male, as risks of depressive symptoms vary significantly between these groups (20% points). In this regard, our analyses can shed a light on the complexity of the intersections of different social strata. By doing so, the results of our analyses contribute to a deeper understanding of inequalities in depressive symptoms. MAIHDA enables new possibilities for analysing social inequalities in depressive symptoms and other (health) outcomes and can identify these differences between different social strata. Examining the variety of intersectional inequalities in depressive symptoms can help identify vulnerable groups affected by multiple disadvantages that can be addressed by targeted intervention programs.

## Electronic supplementary material

Below is the link to the electronic supplementary material.

Supplementary Material 1

## Data Availability

Data is provided within the manuscript or supplementary information files. The related R code is deposited at osf.io (DOI: 10.17605/OSF.IO/C58GT). The study used secondary data from the German National Cohort health study (NAKO), which is available on reasonable request under: https://nako.de/informationen-auf-englisch/.

## References

[1] Hansen T, Slagsvold B, Veenstra M. Educational inequalities in late-life depression across Europe: results from the generations and gender survey. Eur J Ageing. 2017;14(4):407–18. 29180946 10.1007/s10433-017-0421-8 PMC5684038

[2] Niemeyer H, Bieda A, Michalak J, Schneider S, Margraf J. Education and mental health: do psychosocial resources matter? SSM Popul Health. 2019;7:100392. 30989104 10.1016/j.ssmph.2019.100392 PMC6447754

[3] Streit F, Zillich L, Frank J, Kleineidam L, Wagner M, Baune BT et al. Lifetime and current depression in the German National cohort (NAKO). World J Biol Psychiatry. 2023; 24(10): 865-880. 10.1080/15622975.2021.2014152 34870540

[4] Link BG, Phelan J. Social conditions as fundamental causes of disease. J Health Soc Behav. 1995;Spec No:80–94. 7560851

[5] Lorant V, Deliège D, Eaton W, Robert A, Philippot P, Ansseau M. Socioeconomic inequalities in depression: a meta-analysis. Am J Epidemiol. 2003;157(2):98–112. 12522017 10.1093/aje/kwf182

[6] Patil PA, Porche MV, Shippen NA, Dallenbach NT, Fortuna LR. Which girls, which boys? The intersectional risk for depression by race and ethnicity, and gender in the U.S. Clin Psychol Rev. 2018;66:51–68. 29310973 10.1016/j.cpr.2017.12.003

[7] Evans CR, Erickson N. Intersectionality and depression in adolescence and early adulthood: A MAIHDA analysis of the National longitudinal study of adolescent to adult health, 1995–2008. Soc Sci Med. 2019;220:1–11. 30390469 10.1016/j.socscimed.2018.10.019

[8] Glaesmer H, Riedel-Heller S, Braehler E, Spangenberg L, Luppa M. Age- and gender-specific prevalence and risk factors for depressive symptoms in the elderly: a population-based study. Int Psychogeriatr. 2011;23(8):1294–300. 21729425 10.1017/S1041610211000780

[9] Nesterko Y, Friedrich M, Brähler E, Hinz A, Glaesmer H. Mental health among immigrants in Germany – the impact of self-attribution and attribution by others as an immigrant. BMC Public Health. 2019;19(1):1697. 31852465 10.1186/s12889-019-8060-y PMC6921409

[10] Vonneilich N, Becher H, Bohn B, Brandes B, Castell S, Deckert A, et al. Associations of migration, socioeconomic position and social relations with depressive Symptoms – Analyses of the German National cohort baseline data. Int J Public Health. 2023;68:1606097. 37533684 10.3389/ijph.2023.1606097 PMC10391163

[11] Hölzel L, Härter M, Reese C, Kriston L. Risk factors for chronic depression — A systematic review. J Affect Disord. 2011;129(1):1–13. 20488546 10.1016/j.jad.2010.03.025

[12] Brydsten A, Rostila M, Dunlavy A. Social integration and mental health - a decomposition approach to mental health inequalities between the foreign-born and native-born in Sweden. Int J Equity Health. 2019;18(1):48. 30944004 10.1186/s12939-019-0950-1 PMC6889340

[13] Kuehner C. Why is depression more common among women than among men? Lancet Psychiatry. 2017;4(2):146–58. 27856392 10.1016/S2215-0366(16)30263-2

[14] Musliner KL, Munk-Olsen T, Eaton WW, Zandi PP. Heterogeneity in long-term trajectories of depressive symptoms: patterns, predictors and outcomes. J Affect Disord. 2016;192:199–211. 26745437 10.1016/j.jad.2015.12.030 PMC4761648

[15] Close C, Kouvonen A, Bosqui T, Patel K, O’Reilly D, Donnelly M. The mental health and wellbeing of first generation migrants: a systematic-narrative review of reviews. Global Health. 2016;12(1):47. 27558472 10.1186/s12992-016-0187-3 PMC4997738

[16] Elshahat S, Moffat T, Newbold KB. Understanding the healthy immigrant effect in the context of mental health challenges: A systematic critical review. J Immigr Minor Health. 2022;24(6):1564–79. 34807354 10.1007/s10903-021-01313-5 PMC8606270

[17] Aichberger M, Schouler-Ocak M, Mundt A, Busch M, Nickels E, Heimann H, et al. Depression in middle-aged and older first generation migrants in Europe: results from the survey of health, ageing and retirement in Europe (SHARE). Eur Psychiatry. 2010;25:468–75. 20615669 10.1016/j.eurpsy.2009.11.009

[18] Bermejo I, Mayninger E, Kriston L, Härter M. [Mental disorders in people with migration background compared with German general population]. Psychiatr Prax. 2010;37(05):225–32. 20340069 10.1055/s-0029-1223513

[19] Baumgartner JS, Renner A, Wochele-Thoma T, Wehle P, Barbui C, Purgato M, et al. Impairments in psychological functioning in refugees and asylum seekers. Front Psychol. 2023;14:1295031. 38259575 10.3389/fpsyg.2023.1295031 PMC10801113

[20] Vonneilich N, Bremer D, von dem Knesebeck O, Lüdecke D. Health patterns among migrant and Non-Migrant Middle- and Older-Aged individuals in Europe-Analyses based on share 2004–2017. Int J Environ Res Public Health. 2021;18(22):12047. 34831800 10.3390/ijerph182212047 PMC8622058

[21] Cho S, Crenshaw KW, McCall L. Toward a field of intersectionality studies: theory, applications, and praxis. Signs. 2013;38(4):785–810.

[22] Krieger N. Measures of racism, sexism, heterosexism, and gender binarism for health equity research: from structural injustice to embodied Harm-An ecosocial analysis. Annu Rev Public Health. 2020;41:37–62. 31765272 10.1146/annurev-publhealth-040119-094017

[23] Evans CR, Williams DR, Onnela JP, Subramanian SV. A multilevel approach to modeling health inequalities at the intersection of multiple social identities. Soc Sci Med. 2018;203:64–73. 29199054 10.1016/j.socscimed.2017.11.011

[24] Wandschneider L, Miani C, Razum O. Decomposing intersectional inequalities in subjective physical and mental health by sex, gendered practices and immigration status in a representative panel study from Germany. BMC Public Health. 2022;22:683. 35392864 10.1186/s12889-022-13022-1 PMC8991479

[25] Fagrell Trygg N, Gustafsson PE, Månsdotter A. Languishing in the crossroad? A scoping review of intersectional inequalities in mental health. Int J Equity Health. 2019;18:115. 31340832 10.1186/s12939-019-1012-4 PMC6657170

[26] Wamala S, Ahnquist J, Månsdotter A. How do gender, class and ethnicity interact to determine health status? J Gend Stud. 2009;18(2):115–29.

[27] German National Cohort (GNC) Consortium. The German National cohort: aims, study design and organization. Eur J Epidemiol. 2014;29(5):371–82. 24840228 10.1007/s10654-014-9890-7 PMC4050302

[28] Schipf S, Schöne G, Schmidt B, Günther K, Stübs G, Greiser KH, et al. [The baseline assessment of the German National cohort (NAKO Gesundheitsstudie): participation in the examination modules, quality assurance, and the use of secondary data]. Bundesgesundheitsbl. 2020;63(3):254–66. 10.1007/s00103-020-03093-z 32047976

[29] Kuss O, Becher H, Wienke A, Ittermann T, Ostrzinski S, Schipf S, et al. Statistical analysis in the German National cohort (NAKO) - Specific aspects and general recommendations. Eur J Epidemiol. 2022;37(4):429–36. 35653006 10.1007/s10654-022-00880-7 PMC9187540

[30] Wiessner C, Keil T, Krist L, Zeeb H, Dragano N, Schmidt B, et al. [Persons with migration background in the German National cohort (NAKO)-sociodemographic characteristics and comparisons with the German autochthonous population]. Bundesgesundheitsbl. 2020;63(3):279–89. 10.1007/s00103-020-03097-9 32034443

[31] Peters A, Peters A, Greiser KH, Göttlicher S, Ahrens W, Albrecht M, et al. Framework and baseline examination of the German National cohort (NAKO). Eur J Epidemiol. 2022;37(10):1107–24. 36260190 10.1007/s10654-022-00890-5 PMC9581448

[32] Kroenke K, Spitzer RL, Williams JBW. The PHQ-9. J Gen Intern Med. 2001;16(9):606–13. 11556941 10.1046/j.1525-1497.2001.016009606.x PMC1495268

[33] Dragano N, Reuter M, Berger K. Increase in mental disorders during the COVID-19 Pandemic-The role of occupational and financial strains. Dtsch Arztebl Int. 2022;119(11):179–87. 35197188 10.3238/arztebl.m2022.0133 PMC9229580

[34] Manea L, Gilbody S, McMillan D. A diagnostic meta-analysis of the patient health Questionnaire-9 (PHQ-9) algorithm scoring method as a screen for depression. Gen Hosp Psychiatry. 2015;37(1):67–75. 25439733 10.1016/j.genhosppsych.2014.09.009

[35] UNESCO. International Standard Classification Of Education ISCED 1997. 1997.

[36] Schenk L, Bau AM, Borde T, Butler J, Lampert T, Neuhauser H, et al. [A basic set of indicators for mapping migrant status. Recommendations Epidemiol Practice] Bundesgesundheitsbl. 2006;49(9):853–60. 10.1007/s00103-006-0018-4 16927038

[37] van Buuren S, Groothuis-Oudshoorn K. Mice: multivariate imputation by chained equations in R. J Stat Softw. 2011;45:1–67.

[38] D’Agostino McGowan L, Lotspeich SC, Hepler SA. The why behind including Y in your imputation model. Stat Methods Med Res. 2024;33(6):996–1020. 38625810 10.1177/09622802241244608

[39] Moons KGM, Donders RART, Stijnen T, Harrell FE. Using the outcome for imputation of missing predictor values was preferred. J Clin Epidemiol. 2006;59(10):1092–101. 16980150 10.1016/j.jclinepi.2006.01.009

[40] Bell A, Holman D, Jones K. Using shrinkage in multilevel models to understand intersectionality. Methodology. 2019;15(2):88–96.

[41] Hox J, Moerbeek M, van de Schoot R. Multilevel Analysis: Techniques and Applications, Second Edition. 2nd ed. New York: Routledge 2010;392.

[42] Evans CR, Leckie G, Subramanian SV, Bell A, Merlo J. A tutorial for conducting intersectional multilevel analysis of individual heterogeneity and discriminatory accuracy (MAIHDA). SSM Popul Health. 2024;26:101664. 38690117 10.1016/j.ssmph.2024.101664 PMC11059336

[43] Evans CR, Borrell LN, Bell A, Holman D, Subramanian SV, Leckie G. Clarifications on the intersectional MAIHDA approach: A conceptual guide and response to Wilkes and Karimi (2024). Soc Sci Med. 2024;350:116898. 38705077 10.1016/j.socscimed.2024.116898

[44] Brooks ME, Kristensen K, van Benthem KJ, Magnusson A, Berg CW, Nielsen A, et al. GlmmTMB balances speed and flexibility among packages for Zero-inflated generalized linear mixed modeling. R J. 2017;9(2):378–400.

[45] Lüdecke D, Ben-Shachar MS, Patil I, Waggoner P, Makowski D. Performance: an R package for assessment, comparison and testing of statistical models. J Open Source Softw. 2021;6(60):3139.

[46] Lüdecke D. Ggeffects: tidy data frames of marginal effects from regression models. J Open Source Softw. 2018;3.

[47] Bauer GR. Incorporating intersectionality theory into population health research methodology: challenges and the potential to advance health equity. Soc Sci Med. 2014;110:10–7. 24704889 10.1016/j.socscimed.2014.03.022

[48] Wemrell M, Mulinari S, Merlo J. Intersectionality and risk for ischemic heart disease in Sweden: categorical and anti-categorical approaches. Soc Sci Med. 2017;177:213–22. 28189024 10.1016/j.socscimed.2017.01.050

[49] Lee J. Pathways from education to depression. J Cross Cult Gerontol. 2011;26(2):121–35. 21416333 10.1007/s10823-011-9142-1

[50] Bjelland I, Krokstad S, Mykletun A, Dahl AA, Tell GS, Tambs K. Does a higher educational level protect against anxiety and depression? The HUNT study. Soc Sci Med. 2008;66(6):1334–45. 18234406 10.1016/j.socscimed.2007.12.019

[51] Barbek R, Ludecke D, von dem Knesebeck O. Intersectional inequalities in health anxiety: multilevel analysis of individual heterogeneity and discriminatory accuracy in the SOMA.SOC study. Front Public Health. 2024;12. 10.3389/fpubh.2024.1388773 PMC11233522 38989118

[52] Thapar A, Eyre O, Patel V, Brent D. Depression in young people. Lancet. 2022;400(10352):617–31. 35940184 10.1016/S0140-6736(22)01012-1

[53] Bilecen B, Vacca R. The isolation paradox: A comparative study of social support and health across migrant generations in the U.S. Soc Sci Med. 2021;283:114204. 34271369 10.1016/j.socscimed.2021.114204

[54] Morawa E, Erim Y. Acculturation and depressive symptoms among Turkish immigrants in Germany. Int J Environ Res Public Health. 2014;11(9):9503–21. 25222474 10.3390/ijerph110909503 PMC4199032

[55] Aichberger MC, Bromand Z, Rapp MA, Yesil R, Montesinos AH, Temur-Erman S, et al. Perceived ethnic discrimination, acculturation, and psychological distress in women of Turkish origin in Germany. Soc Psych Psych Epid. 2015;50:1691–700. 10.1007/s00127-015-1105-3 26276438

[56] Gkiouleka A, Avrami L, Kostaki A, Huijts T, Eikemo TA, Stathopoulou T. Depressive symptoms among migrants and non-migrants in Europe: documenting and explaining inequalities in times of socio-economic instability. Eur J Public Health. 2018;28(suppl5):54–60. 30476088 10.1093/eurpub/cky202

[57] Bartig S, Koschollek C, Bug M, Blume M, Kajikhina K, Geerlings J, et al. Health of people with selected citizenships: results of the study GEDA Fokus. J Health Monit. 2023;8(1):7–33. 37064418 10.25646/11143 PMC10091045

[58] Vink D, Aartsen MJ, Schoevers RA. Risk factors for anxiety and depression in the elderly: A review. J Affect Disord. 2008;106(1):29–44. 17707515 10.1016/j.jad.2007.06.005

[59] Larsen K, Merlo J. Appropriate assessment of neighborhood effects on individual health: integrating random and fixed effects in multilevel logistic regression. Am J Epidemiol. 2005;161(1):81–8. 15615918 10.1093/aje/kwi017

[60] Wiessner C, Licaj S, Klein J, Bohn B, Brand T, Castell S, et al. Health service use among migrants in the German National Cohort—The role of birth region and Language skills. Int J Public Health. 2024;69:1606377. 38510525 10.3389/ijph.2024.1606377 PMC10952844

[61] Krist L, Bedir A, Fricke J, Kluttig A, Mikolajczyk R. The effect of home visits as an additional recruitment step on the composition of the final sample: a cross-sectional analysis in two study centers of the German National cohort (NAKO). BMC Med Res Methodol. 2021;21(1):176. 34425747 10.1186/s12874-021-01357-z PMC8383386

[62] Moran JK, Jesuthasan J, Schalinski I, Kurmeyer C, Oertelt-Prigione S, Abels I, et al. Traumatic life events and association with depression, anxiety, and somatization symptoms in female refugees. JAMA Netw Open. 2023;6(7):e2324511. 37471088 10.1001/jamanetworkopen.2023.24511 PMC10359962

[63] Dragano N, Reuter M, Greiser KH, Becher H, Zeeb H, Mikolajczyk R, et al. [Socio-demographic and employment-related factors in the German National cohort (GNC; NAKO Gesundheitsstudie)]. Bundesgesundheitsbl. 2020;63(3):267–78. 10.1007/s00103-020-03098-8 32034444

[64] Rice SM, Fallon BJ, Aucote HM, Möller-Leimkühler AM. Development and preliminary validation of the male depression risk scale: furthering the assessment of depression in men. J Affect Disord. 2013;151(3):950–8. 24051100 10.1016/j.jad.2013.08.013

[65] Thibodeau MA, Asmundson GJG. The PHQ-9 assesses depression similarly in men and women from the general population. Pers Individ Differ. 2014;56:149–53.

