# Supplementary material for: Depressive symptoms, education, gender and history of migration - an intersectional analysis using data from the German National Cohort (NAKO)
Source: Int J Equity Health. 2025 Apr 21;24:108. doi: 10.1186/s12939-025-02479-2 (PMC12010676; doi:10.1186/s12939-025-02479-2)

# Supplementary Material “Depressive symptoms, education, gender and history of migration - an intersectional analysis using data from the German National Cohort (NAKO)”

## Comparison of results between imputed and non-imputed data

For the multiple imputation of missing values, we included all terms involved in the interaction including the outcome, but not as additional interaction terms. The only relevant variable from the interaction term with missing values that needed to be imputed was “education” (see tables S1 and S2).

To investigate the impact of the multiple imputation on the results, compared all models and figures based on imputed data and non-imputed data. Results were similar, see the figures S4 and S5 below. Numbers slightly change, but there was no essential change in the results or conclusions.

For the non-imputed data, we found no multiplicative (interaction) effect of strata. However, for imputed data, we found a lower PCV value, possibly indicating a weak interaction effect (see table S3 below).

**S1 Table: Distribution of educational levels, imputed data**

| *Education, NAKO, imputed data* | | | | |
| --- | --- | --- | --- | --- |
| Value | N | Raw % | Valid % | Cumulative % |
| high education | 112,755 | 55.06 | 55.06 | 55.06 |
| low education | 5,540 | 2.71 | 2.71 | 57.77 |
| intermediate education | 86,488 | 42.23 | 42.23 | 100.00 |
| missing values | 0 | 0.00 | - | - |
| total N=204783, valid N=204783 | | | | |

**S2 Table: Distribution of educational levels, non-imputed data including missing values**

| *Education, NAKO, non-imputed data* | | | | |
| --- | --- | --- | --- | --- |
| Value | N | Raw % | Valid % | Cumulative % |
| high education | 102,480 | 50.04 | 55.08 | 55.08 |
| low education | 5,212 | 2.55 | 2.80 | 57.88 |
| intermediate education | 78,375 | 38.27 | 42.12 | 100.00 |
| missing values | 18,716 | 9.14 | - | - |
| total N=204783, valid N=186067 | | | | |

**S3 Table: Comparison of PCV for mixed models, for both models using imputed data and non-imputed data including missing values**

| Data | *PCV* | | | | |
| --- | --- | --- | --- | --- | --- |
|  | Model 1 | Model 2 | Model 3 | Model 4 | Model 5 |
| Imputed | - | 0.09 | 0.19 | 0.62 | 0.87 |
| Non-imputed | - | 0.07 | 0.16 | 0.73 | 0.98 |

**S4 Figure: Ranked predicted probabilities (95% CI) of depressive symptoms, based on MAIHDA logistic regression Model 1; imputed data** *(same figure as in the manuscript)*


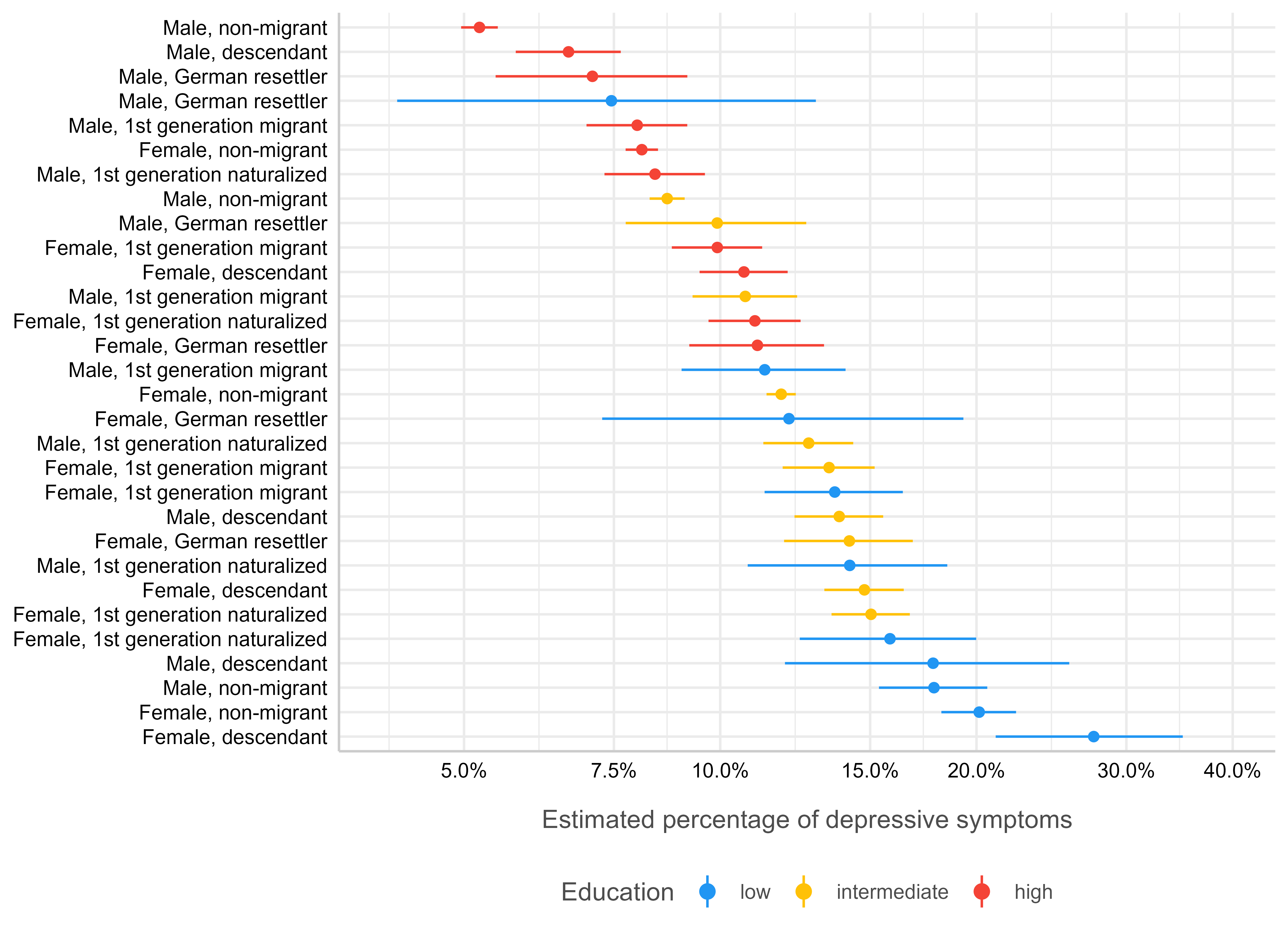


**S5 Figure: Ranked predicted probabilities (95% CI) of depressive symptoms, based on MAIHDA logistic regression Model 1; non-imputed data including missing values**


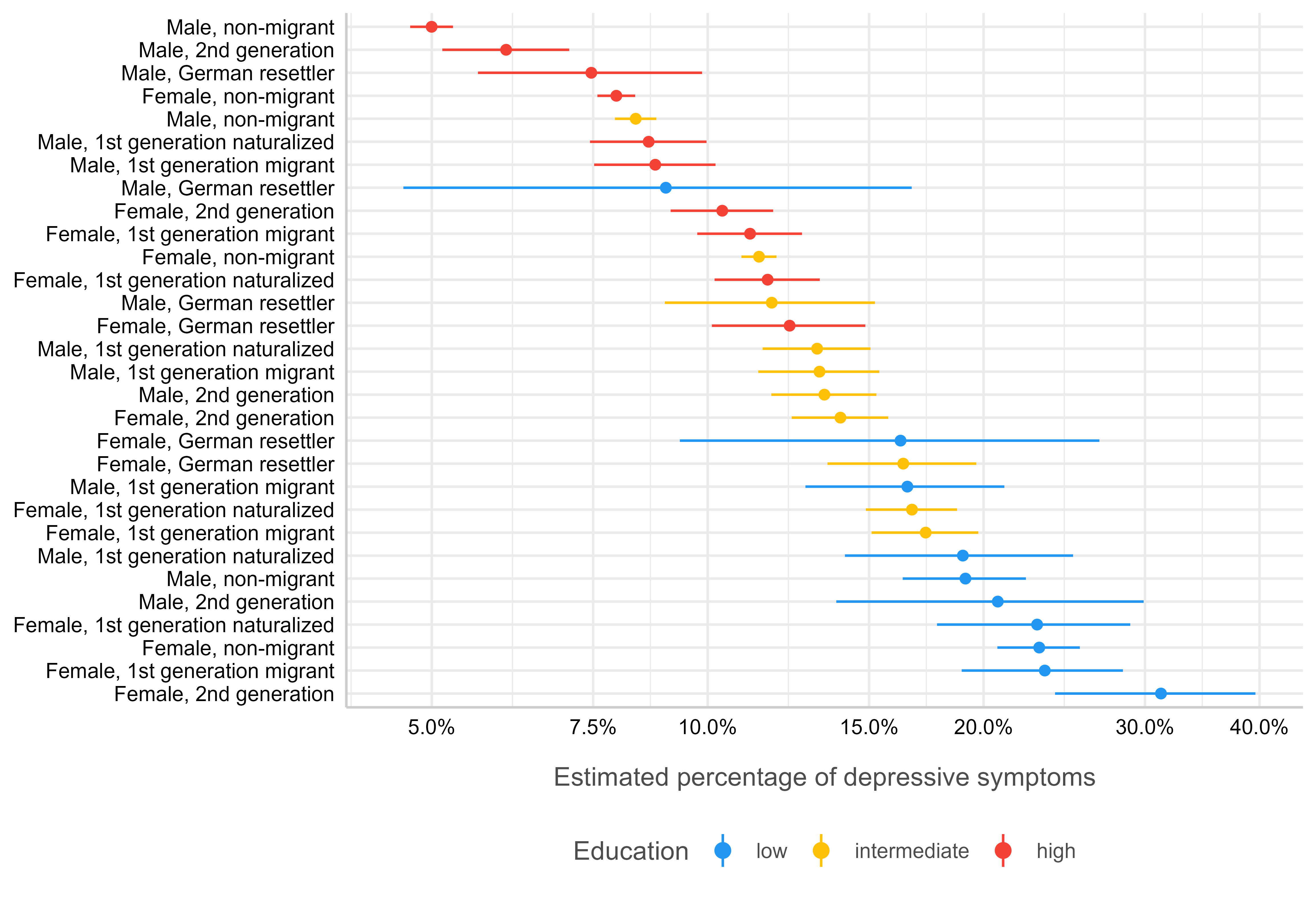


## Performance of logistic regression compared to linear model with log-outcome

For logistic mixed regression models, the proportion of explained variance on the higher levels, as indicated by the ICC, are often rather low. To test whether results would change when running a linear model, we also calculated linear mixed models, using the log of the PHQ-9 score as outcome. The log-transformation is recommended due to the skewed distribution of the outcome (PHQ-9 sum score). Results (in comparison to the results from the logistic regression models) are shown below.

We see very similar patterns, both for the associations between strata and depressive symptoms / score, and for the deviation of strata from the overall mean (cf. figures S4, S6, S7 and S8). This suggests two things: our results regarding intersectionality are most likely no artefact of the logistic regression; multiplicative interaction effects are rather weak, and we find no clear pattern that allows us to make strong conclusions regarding which characteristics are most responsible for such multiplicative effects.

The educational gradient and the differences between male and female persons can be clearly seen for both the logistic and linear regression models, emphasizing that intersectional (not interaction) effects are relevant for our results and conclusions.

**S6 Figure: Ranked predicted probabilities (95% CI) of depressive symptoms, based on MAIHDA linear mixed regression model using log-transormed PHQ-9 Score as outcome; imputed data**

**
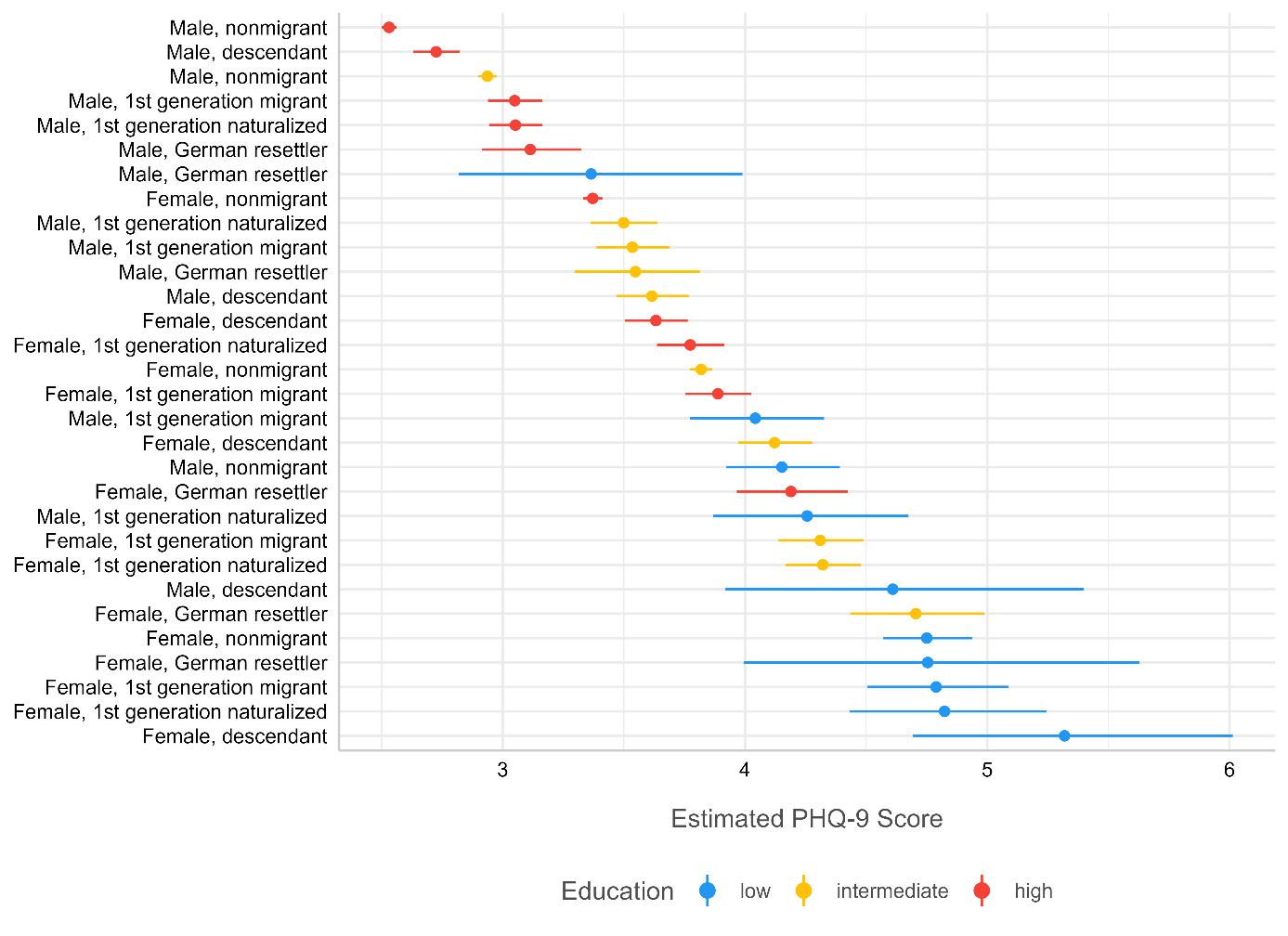
**

**S7 Figure: Predicted stratum random effects (deviation for strata from global mean), full model (logistic mixed model), using PHQ-9 binary as outcome, imputed data**


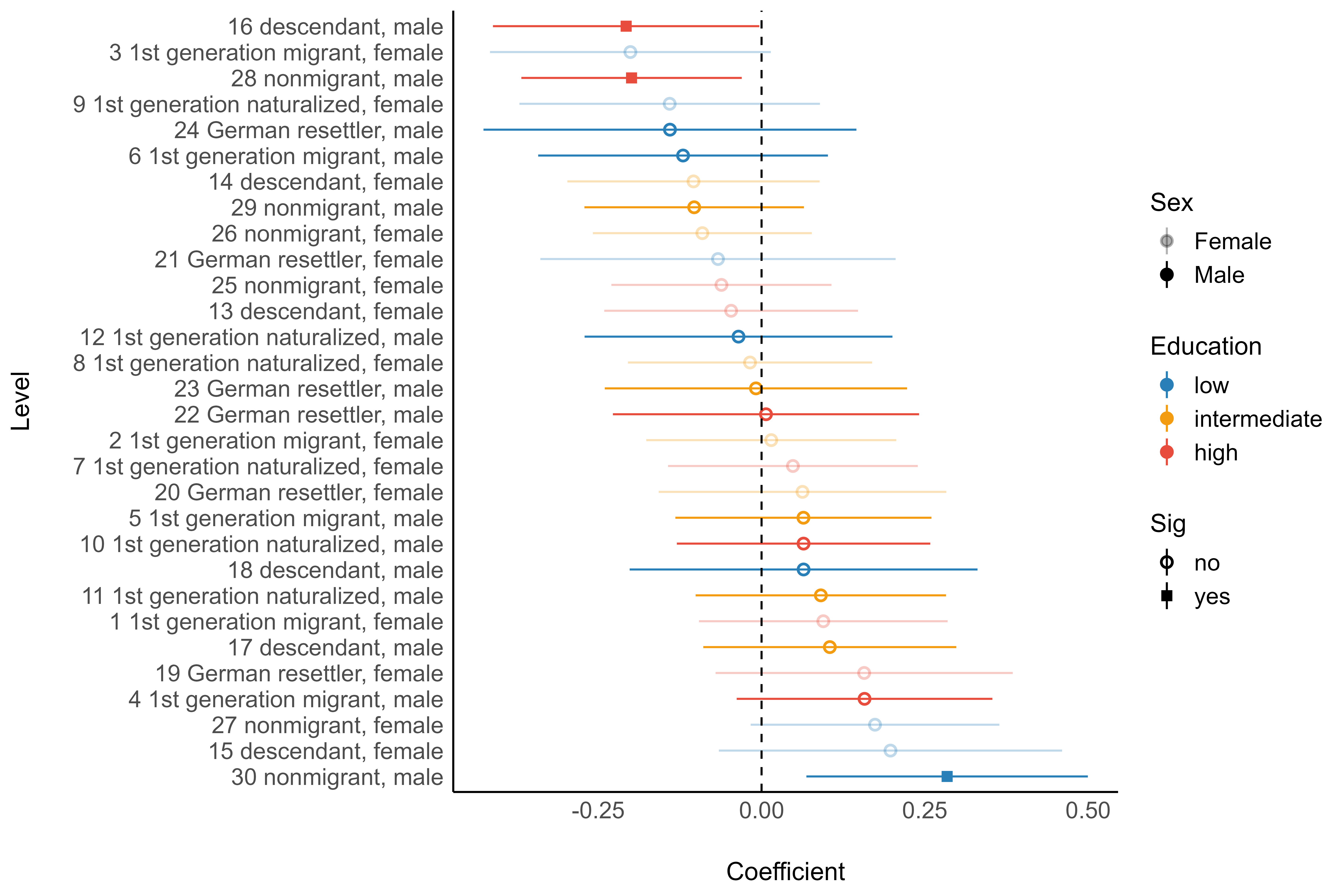


**S8 Figure: Predicted stratum random effects (deviation for strata from global mean), full model (linear mixed model), using log-transformed PHQ-9 as outcome, imputed data**


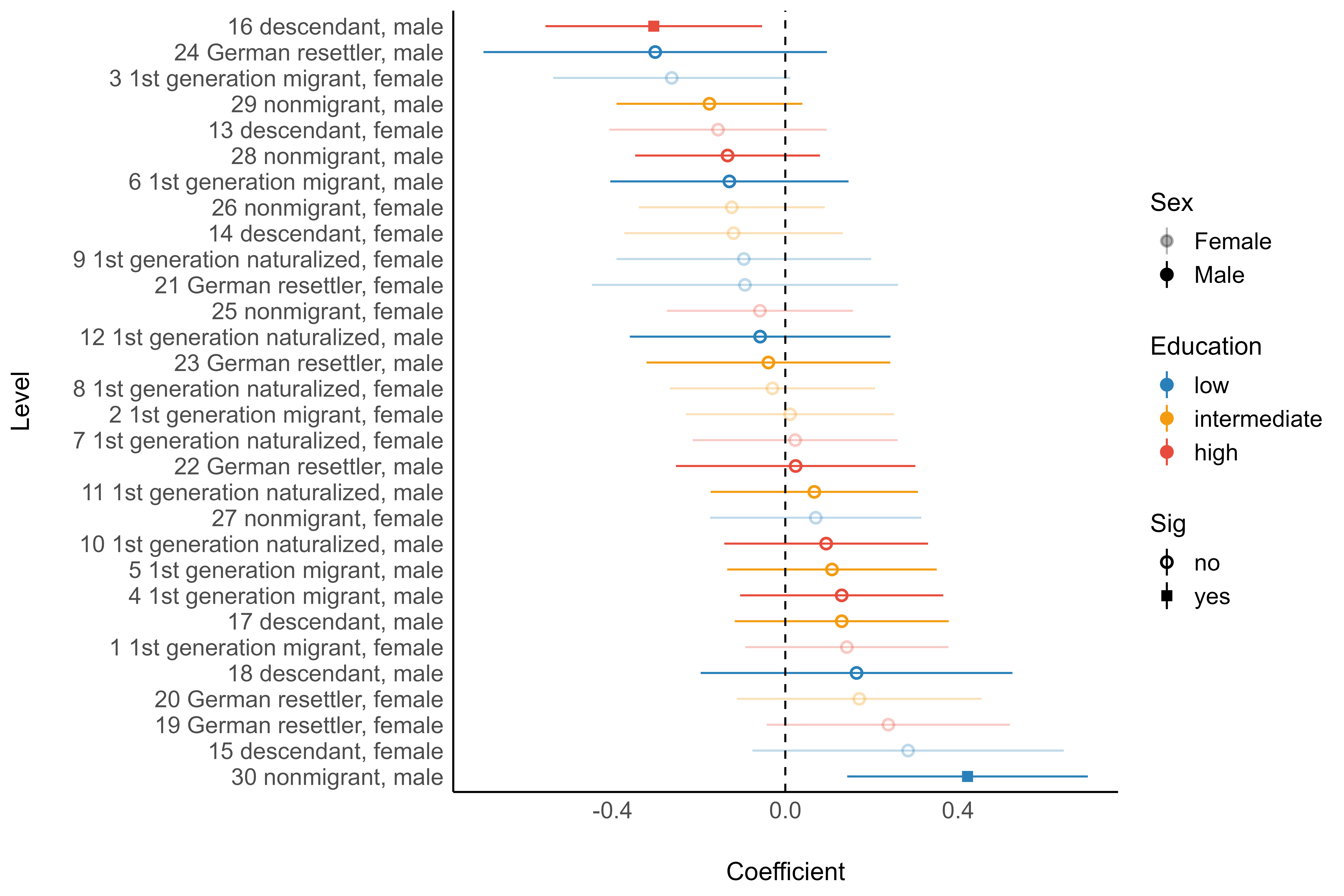

Supplement: Supplementary file 1 — Supplementary Material 1 [file 12939_2025_2479_MOESM1_ESM.docx]
